# Case Report: Leaflet thrombosis after transcatheter valve-in-valve aortic valve replacement in prosthetic valve endocarditis

**DOI:** 10.3389/fcvm.2025.1529523

**Published:** 2025-02-06

**Authors:** Yuhan Zhou, Bo Fu, Nan Jiang, Zhigang Guo

**Affiliations:** ^1^ Clinical School of Thoracic, Tianjin Medical University, Tianjin, China; ^2^ Department of Cardiovascular Surgery, Tianjin Chest Hospital, Tianjin, China

**Keywords:** case report, transcatheter aortic valve replacement, valve-in-valve, leaflet thrombosis, myocardial infarction

## Abstract

A 70-year-old female patient with a history of bioprosthetic aortic valve replacement and coronary artery bypass graft presented with bioprosthetic valve failure secondary to prosthetic valve endocarditis. The patient was deemed unsuitable for surgery by the heart team, following which she underwent transcatheter aortic valve-in-valve replacement. This resulted in early death due to myocardial infarction and acute heart failure. A computed tomography revealed subclinical leaflet thrombosis. This case highlights the importance of postoperative anticoagulation therapy.

## 1 Introduction

A consensus has been reached that all bioprosthetic valves will inevitably fail over time (1). One of the life-threatening complications of surgical aortic valve replacement (SAVR) is prosthetic valve endocarditis (PVE). ESC guidelines (2) recommend early surgery in patients with PVE with heart failure (HF), severe prosthetic dysfunction, abscess, or persistent fever. ACC/AHA guidelines (3) recommend early surgery in patients with PVE who have relapsing infection, while transcatheter aortic valve replacement (TAVR) is an “off-label” treatment, because without complete removal of the infected tissue, the risk of relapse or reinfection will persist. Notably, a recent study showed that 32.5% of patients with infective endocarditis (IE) were considered high risk for surgery (4), making TAVR an effective rescue treatment in select cases or a bridge to SAVR (5–9). However, older patients receiving TAVR are more likely to have prothrombotic comorbidities, and unlike SAVR, which removes native leaflets, TAVR creates neosinuses with the implantation of a transcatheter aortic valve *in situ* (10). This anatomical difference may lead to blood flow stagnation and subclinical leaflet thrombosis (SLT) (11). According to ESC guidelines (12, 13), the first-line antithrombotic therapy is single antiplatelet therapy (SAPT), while oral anticoagulation (OAC) is recommended only if there is an indication such as atrial fibrillation. Furthermore, ACC/AHA guidelines recommend dual antiplatelet therapy (DAPT) or vitamin K antagonist (VKA) in patients with a low risk of bleeding (3). Some studies have reported that patients with SLT managed with anticoagulation regimes, whether with VKA or with non-vitamin K antagonist oral anticoagulants (NOAC), showed regression (14, 15).

## 2 Case presentation

We report the case of a 70-year-old female patient with bioprosthetic valve failure (BVF) and prior PVE complicated by an ascending aortic aneurysm and a coronary artery bypass graft (CABG). The patient underwent a valve-in-valve (ViV) procedure in June 2023. Unfortunately, she required multiple rehospitalizations due to delayed coronary obstruction (CO) and HF and subsequently died. Her medical history is as follows: She underwent SAVR and CABG in May 2006 due to aortic regurgitation (AR) and a 50% stenosis at the ostia of the first diagonal branch. Her native aortic valve was replaced by a 23-mm Medtronic Hancock II bioprosthesis, and she was prescribed oral warfarin for 6 months, followed by life-long aspirin therapy. However, the patient chose to discontinue aspirin because of subcutaneous hemorrhage. In June 2017, she was readmitted due to recurrent symptoms of fatigue, and Holter monitor showed a third-degree atrioventricular block, leading to the implantation of a pacemaker in the following month. Meanwhile, a transthoracic echocardiography (TTE) demonstrated prosthetic aortic valve stenosis with a mean pressure gradient (MPG) of 42 mmHg and a peak velocity (PV) of 4.4 m/s, and the patient declined a redo SAVR. She was followed up annually by TTE; she had an MPG of 21–41 mmHg and a PV of 3.1–4.4 m/s, and the left ventricular ejection fraction (LVEF) declined from 61% in June 2017 to 44% in April 2023. In addition, TTE showed a dilation of the ascending aorta with a stable diameter of 45 mm during follow-up. In October 2022, she presented with a fever and a body temperature of 39.0°C. A blood culture was positive for *Enterococcus faecalis*, and an aortic bioprosthesis vegetation was confirmed using TTE; therefore, she was diagnosed with PVE, which was treated with intravenous daptomycin and ampicillin for 8 weeks. In April 2023, she was readmitted with angina, following which she underwent coronary angiography (CAG), which showed a patency of the saphenous vein graft (SVG) and right coronary artery (RCA). Subsequently, in June, she was admitted for congestive HF symptoms such as exertional dyspnea, orthopnea, and peripheral edema, and the initial treatment plan involved a redo SAVR.

**Figure 1:**
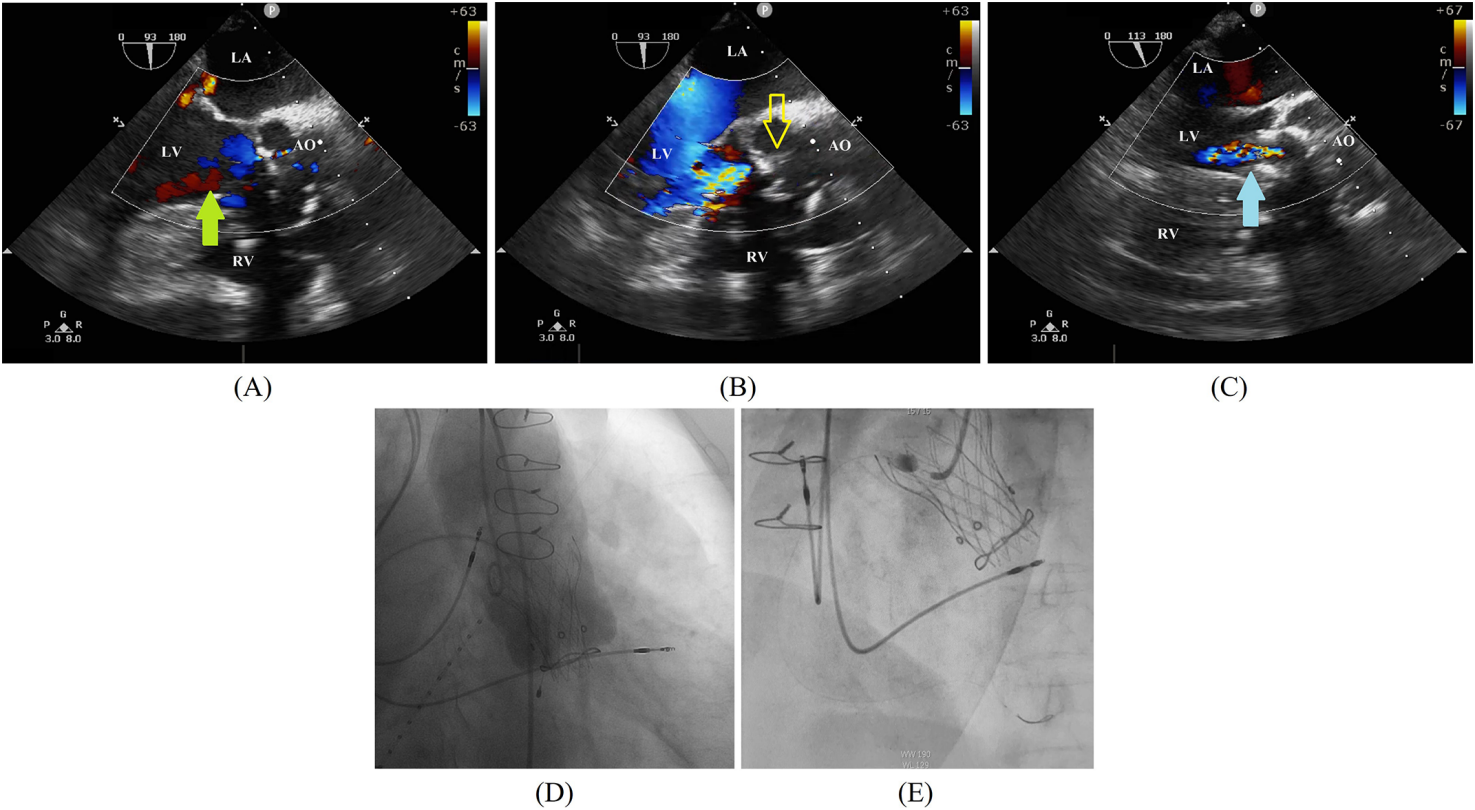
**(A,B)** A preoperative transesophageal echocardiogram demonstrates left coronary sinus perivalvular leakage (green arrow) and a thickened leaflet (yellow outlined arrow). **(C)** A postimplantation transesophageal echocardiogram demonstrates the absence of paravalvular regurgitation. A self-expanding valve bottom skirt fits left ventricular outflow to eliminate perivalvular regurgitation (blue arrow). **(D)** A final angiogram further confirms the absence of valvular or paravalvular regurgitation. **(E)** Coronary angiography demonstrates an obstruction of the proximal RCA. AO, ascending aorta; LA, left atrium; LV, left ventricular; RV, right ventricular.

**Figure 2:**
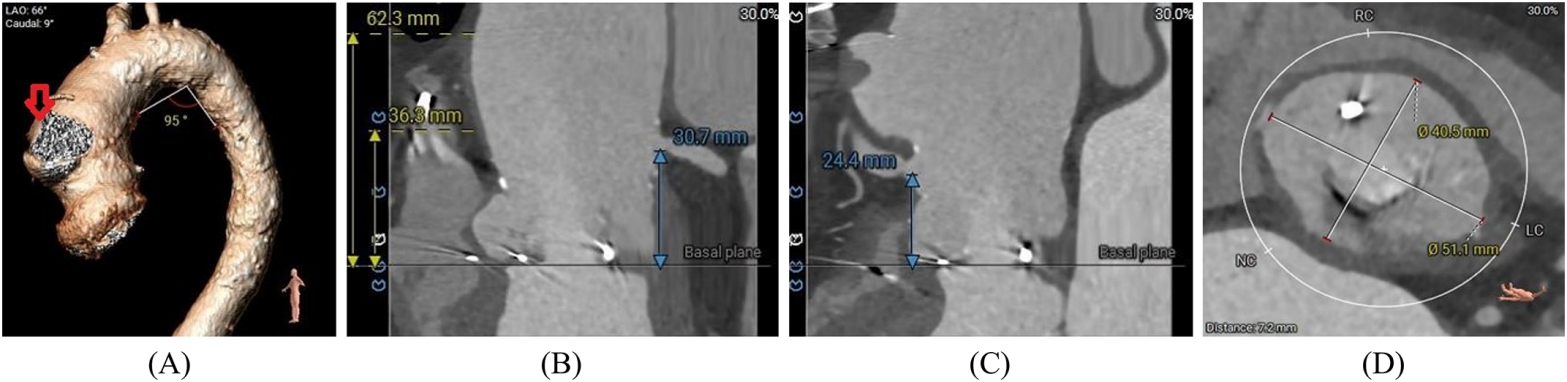
**(A)** Preoperative cardiac-gated CT images were utilized to create a 3D printed model with true-size aortic root dimensions, demonstrating a pseudoaneurysm (red arrow) of the ascending aorta. **(B**,**C)** The distance between the annulus and the ostium of the LCA and RCA. **(D)** The diameter of the sinus of Valsalva.

At admission, the patient's laboratory tests showed the following findings: white blood cell (WBC) count, 6.14 × 10^9^/L; hemoglobin, 111.00 g/L; platelet count, 162.00 × 10^9^/L; blood creatinine, 100.00 μmol/L; estimated glomerular filtration rate, 49.64; aspartate aminotransferase (AST), 13.50 U/L; alanine amino transferase (ALT), 6.90 U/L; and B-type natriuretic peptide (BNP) 469.71 pg/ml. A preoperative transesophageal echocardiogram (TEE) demonstrated thickened leaflets of the prosthetic aortic valve, mild-to-moderate transvalvular regurgitation, mild paravalvular regurgitation, and prosthetic aortic valve stenosis (Figures 1A,B) with an MPG of 23 mmHg, a PV of 3.11 m/s, an left ventricular end-diastolic diameter (LVEDD) of 56 mm, and an LVEF of 43%. TEE also showed moderate mitral regurgitation (MR), mild-to-moderate tricuspid regurgitation (TR), and mild pulmonary hypertension with an estimated pulmonary artery systolic pressure (PASP) of 38 mmHg. A computed tomography (CT) scan showed a patent SVG with a mild stenosis of the left anterior descending artery (LAD) and an ascending aortic aneurysm (Figure 2A). The distances between the annulus and the ostia of the left coronary artery (LCA) and RCA were 30.7 and 24.4 mm, respectively, and the size of the sinus of Valsalva was 40.5 mm × 51.1 mm (Figures 2B–D).

Given the complex anatomy of the aortic root and high surgical risk (STS Score: 8.09%, EuroSCORE II: 32.28%), the patient underwent a ViV procedure with the implantation of a TaurusElite 23-mm self-expandable valve under general anesthesia. A postimplantation TEE demonstrated no paravalvular regurgitation and gradient across the repaired aortic valve with a peak/mean pressure gradient of 30/15 mmHg (Figure 1C). The final angiography further confirmed no transvalvular or paravalvular regurgitation (Figure 1D). Her postoperative courses were uneventful. TTE performed 2 days postoperatively showed a well-functioning transcatheter aortic valve with no transvalvular or paravalvular regurgitation, an MPG of 18 mmHg, a PV of 3.05 m/s, and an LVEF of 47.5%. The patient was discharged after 7 days with a prescription of oral warfarin for at least 3 months. The international normalized ratio (INR) of the patient was 1.2 before discharge. However, the patient did not monitor her INR regularly after discharge, the value of which was not within the therapeutic range. She received follow-up treatment 2 weeks after discharge, and her INR increased to 2.38; subsequently, she was lost to follow-up.

In August 2023, approximately 2 months after surgery, the patient was admitted to the emergency department for angina with ECG findings of ST-segment elevation and a cardiac troponin T level of 5.530 ng/ml. The BNP level was 655.48 pg/ml and the INR was 1.31*.* A TTE demonstrated no dysfunction of the transcatheter aortic valve and an LVEF of 44%. Type 1 myocardial infarction (MI) was determined and an urgent CAG was performed, which showed a complete occlusion of the proximal RCA with thrombolysis in myocardial infarction (TIMI) flow grade 0 (Figure 1E). A coronary thrombus aspiration was done, and a subsequent CAG revealed an occlusion in the distal posterior left ventricular artery (PLA), which was dilated with a balloon. The CAG showed no stenosis in the RCA with TIMI flow grade 3; however, a residual lesion was observed in the distal PLA with TIMI flow grade 0. In addition, a CT scan taken before the CAG showed a patency of the left main stem and left anterior descending artery, a mild stenosis of the circumflex artery, and a patency of the SVG. Two days after the operation, the patient developed dyspnea and elevated central venous pressure; after medical treatment, her symptoms were relieved. She was discharged on day 13 with an SAPT of oral clopidogrel and anticoagulation therapy of oral warfarin. The INR before discharge was 2.89.

In September 2023, approximately 2 weeks after her last hospitalization, she was readmitted to the emergency department for acute HF. At admission, the BNP level was 2,930 pg/ml, and it rapidly increased to over 5,000 pg/ml within hours. The INR was critically high (9.74) with a worsened liver function (AST 385.50 U/L; ALT 311.90 U/L) and a low platelet count of 54.00 × 10^9^/L. A high WBC count of 17.65 × 10^9^/L and procalcitonin level of 6.27 ng/ml also indicated severe infection. Kidney injury was indicated by a high blood creatinine level of 268.00 μmol/L and a potassium level of 5.82 mmol/L. A TTE showed no transvalvular or paravalvular regurgitation of the transcatheter aortic valve with a moderate MR, a moderate-to-severe TR, mild pulmonary hypertension (estimated PASP 38 mmHg), and an LVEF of 38%. Following a preliminary diagnosis of acute HF with infection and multiorgan failure, she was transferred to the intensive care unit. She suddenly developed ventricular fibrillation, and resuscitation was unsuccessful; she was declared clinically dead on Day 19.

### 2.1 Timeline

**Figure 3:**
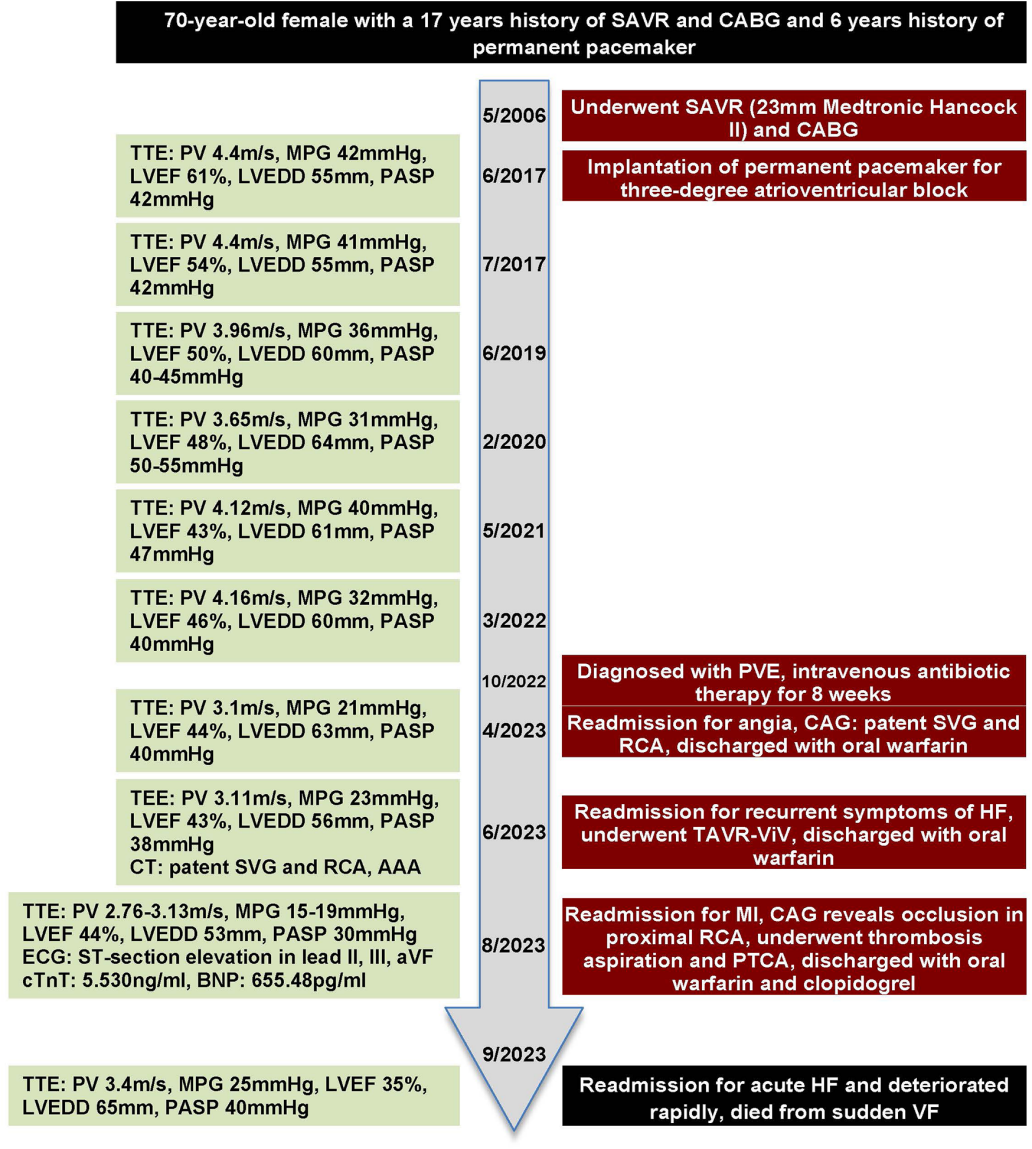
Timeline with key parameters of echocardiography and events throughout this case.

The timeline is shown in Figure 3.

## 3 Discussion

Most patients with BVF are older and usually have comorbidities (1). With studies showing comparable early mortality compared with redo SAVR, a ViV procedure in patients with high or prohibitive surgical risk is supported by the current guidelines (2, 3).

According to the EURO-ENDO registry, only 73.9% of patients with PVE who were indicated for surgery finally underwent one (16). Several case reports have also demonstrated that TAVR can act as a rescue treatment for patients with IE having prohibitive surgical risk and presenting with cardiogenic shock or multiorgan failure (6, 8, 9, 17–20). Moreover, a recent multicenter study showed the feasibility and safety of TAVR in treating “healed” IE with residual AR (5).

Although the patient in this report had stable hemodynamic parameters and no signs of sepsis at admission, the anatomical complexity of the ascending aorta made the surgery considerably challenging; therefore, the heart team decided to perform a ViV procedure. Retrospectively speaking, the risk of CO was considered relatively low due to the fairly safe height of the coronary ostia (>12 mm) and optimal diameter of the sinus of Valsalva (>30 mm) (21) measured by the preoperative CT scan. Moreover, Medtronic Hancock II, as a stented bioprosthetic valve, is not considered a risk factor for CO according to the data from a large multicenter registry (22). However, ViV is associated with a higher risk of CO, which occurs in <1% patients postnative valve TAVR (23, 24) and in 2.3% of patients post ViV (22). Percutaneous coronary intervention (PCI) is attempted in most patients with ST-segment elevation MI after TAVR; however, self-expandable valves present a challenge in terms of coronary access for CAG and PCI. Moreover, without successful revascularization, patients have an increased risk of mortality (25). Although the cause of CO is hard to determine, we suspect a potential thrombotic origin.

SLT is indicated by CT revealing hypoattenuated leaflet thickening (HALT) and restricted leaflet motion (RLM) (26, 27). The reported rates of incidence of SLT range from 11% to 54.1% and vary across studies with different rates of time-to-first detection (14, 28–32). SLT has been a recognized event since Makkar et al. (33) first described it, and CT was mandated in clinical trials by the FDA. However, with the accumulated data from clinical trials and registries, more questions than answers have arisen. First, the time course of SLT remains unclear as its occurrence varies from weeks to months postoperatively. In most clinical trials in the past, the first CT scan was conducted as per protocol at 1 month (28, 29) or 3 months (30, 32) after TAVR; however, in real-world settings, the decision to conduct a CT scan is often left to the discretion of the treating physician. Furthermore, some patients are unsuitable for a CT scan due to comorbidities such as renal dysfunction; hence, serial CT scans after TAVR may not be an ideal modality for the detection of SLT due to repeated exposure of patients to radiation and contrast agents. Second, the clinical significance of SLT is still under debate as patients are asymptomatic. However, valve function evaluated by TTE has yielded conflicting results. For example, a recent retrospective study (34) on the natural history of patients with HALT with a median follow-up time of 4.7 years reported that no patients with HALT were initiated anticoagulation therapy. The authors reported no significant difference in the MPG between the HALT and the no-HALT group of patients, which is consistent with the results of two previous randomized controlled trials (28, 29). However, two other studies (31, 35) with long-term follow-up showed that HALT was significantly associated with increased MPG. Third, whether SLT can be prevented by OAC remains unclear. In the Global Study Comparing a rivAroxaban-based Antithrombotic Strategy to an antipLatelet-based Strategy After Transcatheter aortIc vaLve rEplacement to Optimize Clinical Outcomes (GALILEO-4D) substudy (32), the incidence of HALT was significantly lower in patients receiving an anticoagulation of rivaroxaban with aspirin than in those receiving DAPT. However, in the main trial (36), rivaroxaban-based antithrombotic strategy was associated with an increased risk of thromboembolism complications and major bleeding. In the Anti-Thrombotic Strategy After Trans-Aortic Valve Implantation for Aortic Stenosis (ATLANTIS-4D-CT) substudy (30), apixaban reduced the incidence of HALT or RLM without increasing the risk of thromboembolic or bleeding events. Some authors have suggested that SLT may be spontaneously resolved irrespective of whether OAC therapy is initiated (28, 29). In the current case, although the patient was administered warfarin after TAVR, she eventually developed leaflet thrombosis. With such unanswered questions, we conclude that it is best to personalize anticoagulation therapy and CT scans. The ongoing The Nordic Aortic Valve Intervention-4 (NOTION-4) trial (37), which will randomize patients to anticoagulation or SAPT, may provide insights on HALT after TAVR and its antithrombotic management.

## 4 Conclusion

A ViV procedure may serve as a potential therapy in select patients with PVE; however, the prognosis worsens when leaflet thrombosis leads to CO and MI. TEE is not sensitive enough to detect HALT after TAVR, and a CT scan should be considered when TEE shows increased MPG. SAPT after TAVR is safe but may not prevent the progression of HALT. Given the limited data from long-term follow-up, whether HALT leads to hemodynamic valve deterioration remains unclear.

## Data Availability

The original contributions presented in the study are included in the article/Supplementary Material, further inquiries can be directed to the corresponding author.
